# Genome and machine learning analyses reveal 500-bp promoter chromatin accessibility as a key regulator of gene expression and catechin biosynthesis in *Camellia sinensis*

**DOI:** 10.1093/hr/uhag129

**Published:** 2026-04-06

**Authors:** Yuan Gao, Wenmin Fan, Wenlong Lei, Yibing Chen, Xueli Liao, Yezi Xiao, Lanying Li, Xiaomei Lei, Wanjun Gao, Yingao Zhang, Hongyan Deng, Jiawei Yan, Zongjie Wu, Chen Liang, Xinru Hou, Youben Yu, Fan Luo, Pengjie Wang, Dongna Liu

**Affiliations:** Tea Research Institute, Tea Resources Utilization and Quality Testing Key Laboratory of Sichuan Province, Sichuan Academy of Agricultural Sciences, Chengdu 610066, China; College of Horticulture, Northwest A&F University, Xianyang 712199, China; College of Horticulture, Northwest A&F University, Xianyang 712199, China; College of Information Engineering, Northwest A&F University, Yangling, Shaanxi 712100, China; Tea Research Institute, Tea Resources Utilization and Quality Testing Key Laboratory of Sichuan Province, Sichuan Academy of Agricultural Sciences, Chengdu 610066, China; College of Horticulture, Northwest A&F University, Xianyang 712199, China; Tea Research Institute, Tea Resources Utilization and Quality Testing Key Laboratory of Sichuan Province, Sichuan Academy of Agricultural Sciences, Chengdu 610066, China; College of Horticulture, Northwest A&F University, Xianyang 712199, China; Tea Research Institute, Tea Resources Utilization and Quality Testing Key Laboratory of Sichuan Province, Sichuan Academy of Agricultural Sciences, Chengdu 610066, China; College of Horticulture, Northwest A&F University, Xianyang 712199, China; College of Horticulture, Northwest A&F University, Xianyang 712199, China; College of Horticulture, Northwest A&F University, Xianyang 712199, China; College of Horticulture, Northwest A&F University, Xianyang 712199, China; College of Horticulture, Northwest A&F University, Xianyang 712199, China; College of Horticulture, Northwest A&F University, Xianyang 712199, China; College of Horticulture, Northwest A&F University, Xianyang 712199, China; Tea Research Institute, Tea Resources Utilization and Quality Testing Key Laboratory of Sichuan Province, Sichuan Academy of Agricultural Sciences, Chengdu 610066, China; College of Horticulture, Northwest A&F University, Xianyang 712199, China; Tea Research Institute, Tea Resources Utilization and Quality Testing Key Laboratory of Sichuan Province, Sichuan Academy of Agricultural Sciences, Chengdu 610066, China

Dear Editor,

Tea (*Camellia sinensis*) displays considerable metabolic and agronomic diversity, and high-quality cultivars often exhibit characteristic biochemical profiles that influence tea flavor and quality [1]. However, the regulatory mechanisms underlying these traits, particularly the relationship between chromatin accessibility and gene expression in tea leaves, remain insufficiently understood. Although chromatin openness plays a central role in transcriptional regulation in many plant species, its relative contribution at different genomic positions and its impact on cultivar-specific expression patterns have not been systematically investigated. To establish a framework for regulatory analysis, we generated a high-quality ‘Ganlu 1’ reference genome and integrated chromatin accessibility and transcriptome data from multiple cultivars. Using a supervised machine learning approach, we assessed the contribution of accessible chromatin at distinct genomic positions to gene expression. These analyses further enabled the investigation of promoter accessibility and the transcriptional regulation of genes involved in catechin biosynthesis.

A *de novo* genome assembly of the tea cultivar ‘Ganlu 1’ was generated using 97.33 Gb of PacBio HiFi reads (Fig. 1A and Supplementary Table S1). An initial assembly of 5.20 Gb was produced and subsequently refined by k-mer based filtering using Khaper (https://github.com/lardo/khaper), resulting in a final assembly size of 3.14 Gb. The genome showed 96.3% BUSCO completeness and a contig N50 of 14.9 Mb (Fig. 1B and Supplementary Tables S2 and S3), representing a substantial improvement over representative tea genome assemblies previously reported and widely used in genomic studies [2, 3]. The contigs were anchored using chromatin contact patterns from Hi-C reads and assembled into 15 pseudo-chromosomes, resulting in a chromosome-level assembly (Fig. 1C). The genome assembly assessment showed a long terminal repeat (LTR) assembly index (LAI) of 12.28 (Fig. 1B), indicating high continuity in the assembly. Hi-C contact heatmaps and synteny analysis further confirmed the structural consistency and accuracy of the assembled genome (Supplementary Figs S1 and S2). Protein-coding genes were annotated by integrating transcriptomic evidence, homologous protein sequences, and *de novo* predictions, resulting in the identification of 59 946 genes (Supplementary Table S4). Functional assignments across Pfam, Clusters of Orthologous Groups (COG), Kyoto Encyclopedia of Genes and Genomes (KEGG), and Gene Ontology (GO) databases produced a high-confidence annotated gene set, as summarized in the UpSet plot (Supplementary Fig. S3). Repetitive sequences occupied a major proportion of the genome, with LTR retrotransposons representing the dominant class (Supplementary Table S6).

**Figure 1:**
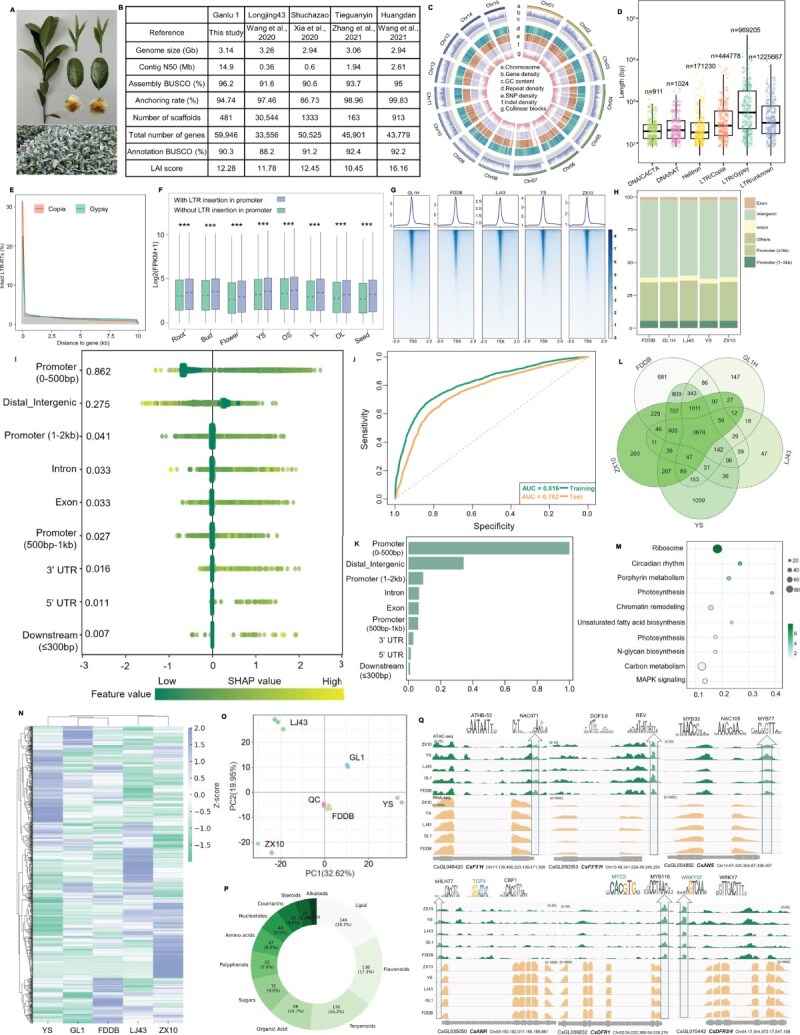
Multi-omics integration reveals promoter accessibility patterns in tea. (A) Representative whole-plant morphology and tissue phenotypes of the ‘Ganlu 1.’ (B) Comparison of the ‘Ganlu 1’ genome with other tea plant genomes. (C) Circos plot representing the genomic features of the ‘Ganlu 1’ genome (SNP and Indel densities were calculated relative to the ‘Fuding Dabaicha’ reference genome). (D) Length distribution of major TE superfamilies. (E) Distribution of the distance of tea plant LTR-RTs to protein-coding genes. Only distances %10 kb are plotted. (F) Effect of LTR insertions in promoter regions on gene expression across multiple tissues, including root, bud, flower, young stem (YS), old stem (OS), young leaf (YL), old leaf (OL), and seed. (G) Average plots and heat maps of signals at the OCRs. (H) Genomic distribution of OCRs across cultivars, including ‘Fuding Dabaicha’ (FDDB), ‘Ganlu 1’ (GL1H), ‘Longjing 43’ (LJ43), ‘Yusun’ (YS), and ‘Zhongxuan 10’ (ZX10). (I) Feature ranking for predicting gene expression using a supervised XGBoost model trained on OCRs located at different genomic positions. (J) ROC curves and AUC values of the XGBoost model. (K) Feature importance of the XGBoost model based on OCR related features. (L) Venn diagram of 1-kb promoter OCR of the five cultivars. (M) KEGG pathway enrichment analysis of genes associated with the overlapping 1-kb promoter OCRs shared by all five cultivars. (N) Untargeted metabolomic analysis of the five cultivars. (O) PCA of untargeted metabolomic profiles across the five cultivars. (P) Composition of untargeted metabolite classes. (Q) Genome browser views showing transcript abundance and promoter (1 kb) OCRs for six key genes involved in catechin biosynthesis. OCRs in the promoter regions were annotated with high-confidence transcription factor (TF) binding motifs (*P* ≤ 1E − 4) predicted using FIMO and SEA. Experimentally validated TFs are highlighted in the figure.

Comprehensive annotation of transposable elements (TEs) revealed that repetitive sequences constitute a major portion of the *Camellia sinensis* genome [1]. LTR retrotransposons (LTR-RTs) represented the dominant TE class, with *Gypsy* (36.22%), *Copia* (7.44%), and unclassified LTR elements (21.91%), accounting for the largest fractions of the genome based on cumulative sequence length (Supplementary Fig. S4). The length distributions of the major TE superfamilies further illustrated substantial differences among DNA transposons, Helitrons, and LTR-RTs (Fig. 1D), highlighting the extensive expansion of LTR elements in the tea genome. Interestingly, a large proportion of intact LTR-RTs were located within 2 kb upstream of protein-coding genes (Fig. 1E and Supplementary Table S5). Consistent with this expectation, genes with LTRs inserted into their promoter regions showed significantly higher expression levels in various tissues than genes lacking promoter-associated LTRs (Fig. 1F). Together, these observations indicate a significant association between promoter-proximal LTRs and gene expression levels in tea plants.

Based on the high-quality ‘Ganlu 1’ reference genome generated in this study, chromatin accessibility profiles were further examined across five tea cultivars using ATAC-seq (Fig. 1G). Genome-wide annotation revealed that open chromatin regions (OCRs) were predominantly located within promoter-proximal and intergenic regions across all cultivars, including ‘Fuding Dabaicha’ (FDDB), ‘Ganlu 1’ (GL1H), ‘Longjing 43’ (LJ43), ‘Yusun’ (YS), and ‘Zhongxuan 10’ (ZX10) (Fig. 1H and Supplementary Fig. S8). To explore cultivar-dependent regulatory variation, cultivar-specific accessible chromatin regions (ACRs) were further characterized. The length distribution of unique ACRs differed among cultivars (Supplementary Fig. S5), and their abundance also displayed considerable variation (Supplementary Fig. S6), indicating substantial differences in regulatory element usage and chromatin accessibility across the tea cultivars examined. Moreover, within each cultivar, genes associated with cultivar-specific ACRs exhibited significantly higher expression levels than other genes (Supplementary Fig. S7), supporting a consistent association between chromatin accessibility and transcriptional activity across cultivars.

To quantify the relative contribution of chromatin accessibility at different genomic positions to gene expression, and to enable a unified comparison of accessibility effects across multiple genomic contexts, a supervised XGBoost model was trained using OCR features stratified by genomic region (Supplementary Tables S7 and S8). SHAP interpretation revealed that OCRs located within the proximal promoter had the highest predictive importance (0.862), with the 0- to 500-bp region upstream of the transcription start site exhibiting the highest contribution, markedly exceeding the contributions of distal intergenic, intronic, exonic, UTR, and downstream OCRs (Fig. 1I). This result emerged from a stratified positional analysis of upstream regions, and indicates that promoter-proximal accessibility, particularly within the first 500-bp upstream of genes, is the strongest predictor of gene expression among all assessed genomic contexts. Model performance evaluation using receiver operating characteristic (ROC) curves demonstrated robust predictive accuracy, with high area under the curve (AUC) values (Fig. 1J). The feature importance hierarchy further confirmed the dominant regulatory role of promoter-localized OCRs, particularly those in the proximal promoter, in determining gene expression levels (Fig. 1K). To assess the conservation of promoter accessibility across cultivars, proximal promoter-associated OCRs were compared among the five samples. A small but clearly defined set of promoter ACRs was shared across all cultivars (Fig. 1L and Supplementary Table S9). KEGG enrichment of genes associated with these overlapping promoter ACRs revealed significant enrichment in transcription- and metabolism-related pathways (Fig. 1M), indicating that a conserved core of promoter-accessible regions is preserved across tea cultivars to maintain essential biological functions.

Untargeted metabolomic profiling was performed for five tea cultivars to characterize their metabolite composition (Fig. 1N). Principal component analysis (PCA) revealed clear multidimensional structure within the dataset, indicating substantial variation in metabolite abundance patterns (Fig. 1O). Metabolites were classified into several major chemical categories, with lipids, flavonoids, organic acids, terpenoids, amino acids, nucleotides, polyphenols, and sugars representing the predominant classes detected across samples (Fig. 1P and Supplementary Fig. S9). It is noteworthy that flavonoids and polyphenols, which include catechins and their related derivatives, accounted for a significant proportion of the detected metabolites, highlighting the importance of catechin-related metabolic pathways in tea.

Catechins constitute a major group of bioactive metabolites in tea leaves. Among them, (−)-epigallocatechin-3-gallate (EGCG) is the most abundant and plays an important role in the quality of tea [4]. To investigate the transcriptional regulation underlying catechin biosynthesis, chromatin accessibility and transcriptome data were analyzed for six key structural genes in this pathway, including flavonoid 3′-hydroxylase (F3′H), flavonoid 3′,5′-hydroxylase (F3′5′H), anthocyanidin synthase (ANS), anthocyanidin reductase (ANR), and the dihydroflavonol 4-reductase paralogs (DFR1 and DFR3/4). These enzymes act at multiple branch points of the flavonoid pathway and directly influence the formation of esterified catechins [5]. Genome browser visualizations showed a clearly detectable OCR within the proximal promoter of representative gene, together with their corresponding transcript abundance profiles (Fig. 1Q). These promoter-proximal OCRs were used as input sequences for motif prediction, and a total of 14 high-confidence transcription factors (TFs) were identified based on FIMO and SEA analyses (*P* ≤ 1E–4) (Supplementary Table S10). Among these predicted regulators, *TCP3*, *MYC2*, and *WRKY57* were inferred to bind the promoters of *CsANR*, *CsDFR1*, and *CsDFR3/4*, respectively. These transcription factors have previously been experimentally validated to regulate these key genes involved in flavonoid and catechin biosynthesis [5]. Additional transcription factors were also identified for each gene, suggesting that more than one regulator may interact with these promoter-proximal accessible regions. Overall, these findings underscore the importance of chromatin accessibility within the proximal promoter region in facilitating transcription factor binding and supporting the transcriptional activation of catechin biosynthetic genes.

The high-quality ‘Ganlu 1’ reference genome generated in this study provides an essential foundation for dissecting transcriptional regulation in tea. Comprehensive analyses indicated that promoter-proximal chromatin accessibility, particularly within the 0- to 500-bp upstream of transcription start sites, is a key structural feature that enables transcription factor binding and facilitates the expression of core metabolic genes. These findings offer a valuable genomic and epigenomic framework for future functional studies and for advancing molecular breeding aimed at improving tea quality.

## Data Availability

The data that support the findings of this study, including genome sequencing, ATAC-seq, and RNA-seq datasets, are publicly available in the National Genomics Data Center (https://ngdc.cncb.ac.cn/) under project number PRJCA052192. The genome assembly and annotation have been deposited in Figshare (https://doi.org/10.6084/m9.figshare.30800579), and the scripts used for XGBoost modeling and OCR analysis are available on GitHub (https://github.com/fwm0624/XGBoost.R). Supplementary figures and tables are available at GitHub (https://github.com/fwm0624/supplementary).
